# Novel Fluorescent Nanocellulose Hydrogel Based on Nanocellulose and Carbon Dots for Detection and Removal of Heavy Metal Ions in Water

**DOI:** 10.3390/foods11111619

**Published:** 2022-05-30

**Authors:** Jiachuan Yang, Zhixin Luo, Min Wang

**Affiliations:** College of Food Science and Engineering, Northwest A&F University, Xianyang 712100, China; 2018060370@nwafu.edu.cn (J.Y.); 2018060369@nwafu.edu.cn (Z.L.)

**Keywords:** nanocellulose, fluorescent nanocellulose hydrogel, heavy metals ion, adsorption, fluorescent sensor

## Abstract

Water is an important raw material in the food production process. Maintaining the quality and safety of water is very important in the food field. In this study, a simple novel fluorescent nanocellulose hydrogel (FNH) was prepared for the detection and removal of heavy metals (Fe^3+^ and Pb^2+^) in aqueous solutions based on carbon dots (CDs). The CDs were grafted onto the carboxylated nanocellulose (CNC) by the EDC/NHS coupling method, and then the nanocellulose (NC), CNC, and FNH were characterized by FTIR analysis. The effect of adsorption environment on FNH adsorption capacity was also investigated. After carboxylation and grafting of CDs, the adsorption capacity of nanocellulose to Fe^3+^ and Pb^2+^ was greatly improved, and it was also allowed to make fast visual responses to Fe^3+^ as an optical sensor to determine the concentration of Fe^3+^ through the visual signal. Static adsorption experiment demonstrated that the removal rate of Fe^3+^ and Pb^2+^ by FNH exceeded 69.4% and 98.2%, and the adsorption capacity amount reached 98.3 mg/g and 442.0 mg/g. At the same time, due to the fluorescence quenching effect of Fe^3+^, FNH could also be used for the detection of Fe^3+^ concentration in aqueous solution, and the limit of detection (LOD) could reach 62.5 mg/L.

## 1. Introduction

Water plays a very crucial role in the field of food. Water is an important raw material for most foods, especially in beverages and similar products, where water occupies an absolute position. Moreover, water is an important agent in food processing. The solubilization of many raw materials (water-soluble substances such as sugars, flavoring agents, etc.) needs water, as well as many food processing processes (high-temperature sterilization, cleaning, etc.). Furthermore, the amount of water will also directly or indirectly affect the quality of the product, which is crucial in the field of food production and processing.

Water quality is very important in the food industry, especially in the preparation of beverages. With the improvement of living standards, people’s worries about food environmental safety are also increasing, especially worries about heavy metals in water, such as cadmium, lead, arsenic, and mercury. Therefore, ensuring the quality and safety of water and removing toxic and harmful substances in water has become an important issue in the field of food industry. Heavy metals are difficult to degrade and easily accumulate in organisms, and their enrichment through the food chain threats human health [1,2]. The most commonly used methods for removing heavy metals in wastewater include biological treatment, chemical precipitation, ion exchange, membrane filtration, and adsorption [3,4].

Nanocellulose (NC), has unique biological adsorption characteristics and has obvious potential for the development of new green heavy metal removal technology [5,6]. The adsorption method has the advantages of good reusability and simple operation, and is currently considered to be the most effective and environmentally friendly method for removing heavy metals in wastewater [7,8]. Due to the various functional groups and three-dimensional network structure of hydrogel, it is considered to be the most promising adsorbent for removing heavy metals in wastewater [9,10]. The combination of nanofibers and hydrogels creates a more favorable three-dimensional network structure and provides a better aggregate for heavy metal adsorption [11,12,13].

In recent years, carbon dots (CDs) have been widely used due to its unique luminescence, chemical stability, biocompatibility, and non-toxicity. At the same time, CDs also have the characteristics of easy access to raw materials and simple production process. Therefore, CDs have been widely used in biological/chemical sensors, biomedical imaging, optoelectronic equipment [14].

In this paper, a novel nanocellulose hydrogel with CDs was prepared for the detection and removal of Fe^3+^ and Pb^2+^ in water. The structure and optical properties of FNH was characterized, and the effect of the adsorption environment (contact time, pH value, and adsorbent dosage) on the adsorption capacity of FNH was investigated to obtain higher adsorption capacity. And the experiments conducted under the same experimental conditions showed that the Fe^3+^ and Pb^2+^ in water adsorption capacity of FNH were significantly higher than that of conventional adsorbents. In addition, FNH was used to determine Fe^3+^ based on the fluorescence quenching effect of Fe^3+^ and CDs.

## 2. Materials and Methods

### 2.1. Materials

1-(3-Dimethylaminopropyl)-3-ethylcarbodiimide hydrochloride (EDC, 98.0%), N-hydroxysuccinimide (NHS, 98%), and N,N-methylenebisacrylamide (MBA, 99%) were obtained from Aladdin Reagent Co., Ltd. (Shanghai, China). Sodium hydroxide, sodium periodate, hydrochloric acid, acetic acid (HAc), sodium acetate (NaAc), acrylamidem and potassium persulfate (PPS, 99.0%) were supplied by Sinopharm Chemical Reagent Company (Shanghai, China). All other chemicals used in this work were of analytical grade.

### 2.2. Preparation of Nanocellulose

Acid hydrolysis and oxidation is mainly used to prepare cellulose nanocrystals obtained by destroying the amorphous region of cellulose. Strong acid, such as sulfuric acid, can easily hydrolyze the amorphous region of cellulose and even leads to carbonization. Therefore, employing weak acids, such as acetic acid, has become a new method for the preparation of NC due to their relatively safe use, high yield, and short preparation time. The cellulose was mixed with 0.5% hydrochloric acid and 88% acetic acid at a solid to liquid ratio of 1:30. Next, the mixture was stirred at 268 K for 1 h. The reaction was terminated by cooling to room temperature with tap water, then centrifuged at 6000 rpm for 5 min to remove excess acid, resuspended with deionized water, and repeat three times. Finally, nanocellulose (NC) was obtained after freeze-dried.

### 2.3. Synthesis of Carboxylated Nanocellulose (CNC)

Sodium periodate and ammonium persulfate oxidation are two commonly used methods to prepare carboxylated nanocellulose. The two methods were combined in this paper to obtain better carboxylation effect [15,16]. The preparation of sodium periodate carboxylated nanocellulose was according to Liu [17], the cellulose was uniformly dispersed in a deionized water solution containing 5% NaIO_4_ and 7.6% CaCl_2_, adjusted the pH to 3.5 and reacted for 6 h at 318 K. The reaction was terminated by adding ethylene glycol [18], followed by washing, three times, with deionized water, and centrifugation. Then, the ammonium persulfate was used for further carboxylated [19,20], and the NC was dispersed in 2 M ammonium persulfate solution, sonicated at 333 K for 6 h, and mixed with 200 mL of 1 M citric and continued sonication for 4 h. The carboxylated NC was washed three times with deionized water and centrifuged. Finally, the CNC was obtained after freeze-dried.

### 2.4. Preparation of CDs

The preparation of CDs was according to previous studies [21,22]. Briefly, 1.15 g citric acid and 33.5 μL ethylenediamine was fully dissolved in 20 mL ultrapure water by sonication, then the prepared mixture was poured into a polytetrafluoroethylene lining and put into an autoclave, kept it in an environment of 573 K for 14 h to complete the hydrothermal reaction, and cooled to room temperature after the reaction is complete. The supernatant was sucked and filtered with 0.45 μm and 0.22 μm water-based filters in sequence to obtain yellow carbon quantum dot solution.

### 2.5. Preparation of Carboxylated Nanocellulose Modified with Carbon Dots (CNC-CDs)

EDC/NHS coupling method was used to connect CDs to CNC [23,24]. Briefly, 0.4 g CNC was fully suspended in 50 mL NaAc/HAc buffer (1 mM, pH 4.5) by sonication, Then, EDC (119 mg), NHS (460 mg), and CDs (30 mg, 0.01 wt%) were added under stirring conditions for 15 min. The reaction was conducted overnight in the dark. The mixture was then dialyzed in a dialysis bag (12,000–14,000 Da) in ultrapure water for 4 days.

### 2.6. Preparation of Fluorescent Nanocellulose Hydrogel (FNH)

The nanocellulose hydrogel with strong fluorescence and high absorption was prepared by free radical polymerization method [25]. Briefly, 2.5 mL of acrylic acid was added to NaOH (5.5 mL, 5 M) with stirring at 273 K, then added with CNC-CDs, acrylamide (0.5 g) and MBA (0.03 g), stirred for 30 min, then added with potassium persulfate and stirred for 30 min. The reaction system was transferred to a water bath at 343 K for 5 h to ensure the reaction complete [26].

### 2.7. Physicochemical Characterization

The presence of active groups in the NC, CNC and nanocellulose hydrogel were obtained by a FTIR spectrometer (Vertex 70, Bruker, Rheinstetten, Germany) in range of 500–4000 cm^−1^, and 20 scans for each sample were conducted. The morphological structure of the different FNH was observed by scanning electron microscope (SEM) (S-4800, Hitachi, Tokyo, Japan), and Nano Measurer was used to measure the length and width of FNH. UV-Vis spectra and fluorescence features of CDs were observed using a microplate reader (Spark, Tecan Austria GmbH, Grödig, Austria).

### 2.8. Adsorption Studies

The adsorption capacity of FNH for heavy metal ions was studied by batch experiments [27,28]. NC and FNH of certain amounts were added to Fe^3+^ and Pb^2+^ solution (50 mg/L, 200 mL) in a 200 mL conical flask, and sampled at different time points, then diluted with ultrapure water (0, 15, 30, 60, 105, 150, and 210 min). The concentrations of Fe^3+^ and Pb^2+^ in the samples were measured by Atomic Absorption Spectrophotometer (Jena Analytical Instruments AG, Jena, Germany) to determine the equilibrium adsorption time. The pH (from 3 to 7) and adsorbent dosage (from 0.15 to 2.4 g) conditions were then determined using the same method. The adsorption isotherm experiments with initial concentrations ranging from 6.25 mg/L to 200.0 mg/L were carried out at 288, 298, and 308 K, respectively.

## 3. Results and Discussion

### 3.1. Physicochemical Characterization

#### 3.1.1. FTIR

**Figure 1:**
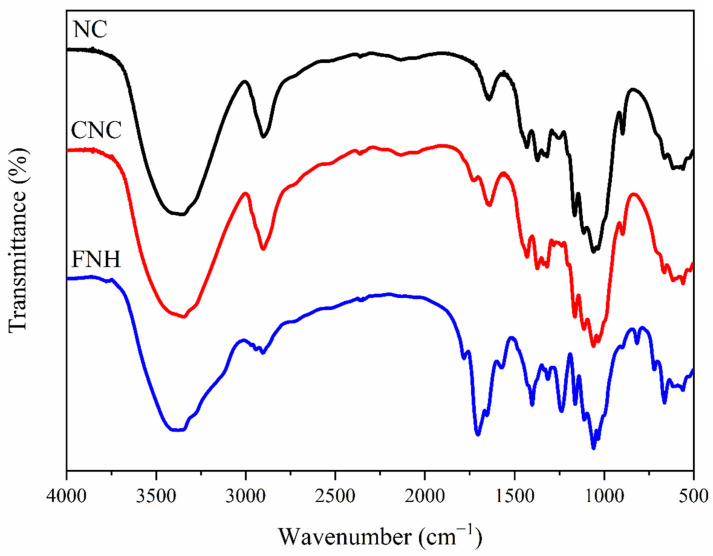
FTIR spectrum of NC, CNC and FNH.

Figure 1 shows the FTIR spectrums of NC, CNC, and FNH. The peaks appearing at 3415 cm^−1^ and 2914 cm^−1^ were attributed to stretching vibrations of O-H and C-H, respectively [29]. The peak at 1052 cm^−1^ was attributed to the C-O-C pyranose ring vibration. In the FTIR spectrum, a band at 1750 cm^−1^ (-CO-) appeared after the NC was carboxylated, which proved that the -COOH was generated on NCs [30]. Compared to the spectrum of CNC, a new band at 1650 cm^−1^ (-CO-NH-) appeared on CNC-CDs, and the 1570 cm^−1^ band was increased in the spectrum of CNC-CDs, which indicated that the CDs was grafted on the CNC [31].

#### 3.1.2. SEM

**Figure 2:**
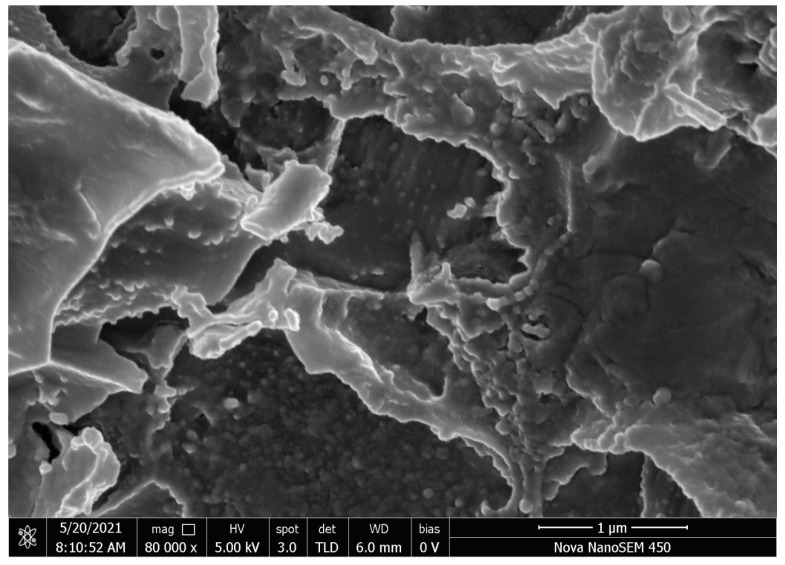
SEM images of FNH.

Figure 2 shows that the SEM image of FNH. FNH has a porous three-dimensional structure, which can provide a high specific surface area for the absorption of heavy metal ions [32].

#### 3.1.3. Spectral Features

**Figure 3:**
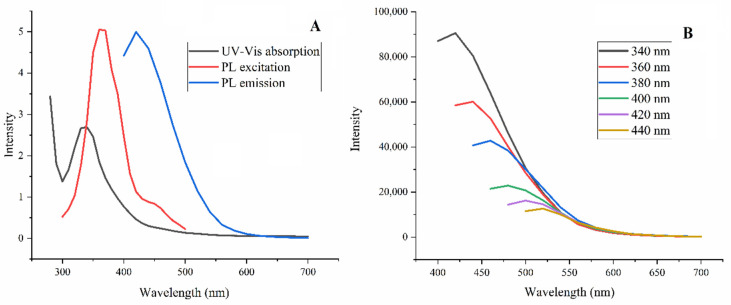
Spectral features of CDs. UV-Vis and PL excitation and emission spectra of CDs (**A**), and excitation-dependent PL spectra (**B**).

Figure 3 shows the UV-Vis spectrum of CDs. There was a distinct characteristic peak at 340 nm, which was mainly attributed to the *n*-π* transition of the carbonyl group in CDs. The photoluminescence (PL) excitation and emission spectra of CDs were also shown in Figure 3A, as could be seen at excitation and emission wavelengths for CDs were 360 nm and 420 nm. Furthermore, as the excitation wavelength increased (from 340 nm to 440 nm), the excitation wavelength deviated from the excitation wavelength, and the fluorescence intensity also decreased (Figure 3B). At the same time, the PL emission peak shifted to long wavelengths with increasing excitation wavelength, which might be caused by the presence of carbonyl and carboxyl.

### 3.2. Appearance and Fluorescence Behavior of FNH

**Figure 4:**
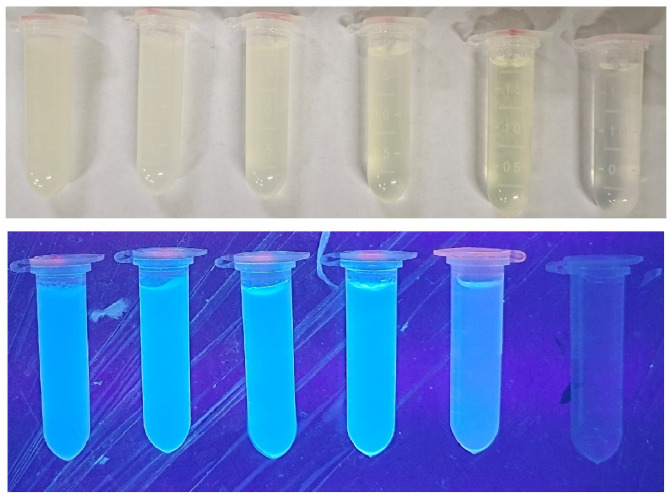
Appearance and fluorescence behavior of synthesized fluorescent nanocellulose hydrogel (FNH).

Figure 4 demonstrates the appearance and fluorescence behavior of FNH (the CNC-CDs content varying from 0.06 to 0.12, 0.25, 0.50, 1.00, and 0 wt%). With the increase of CNC-CDs quality, the fluorescent brightness was enhanced. Among these hydrogels, 0.50 wt% showed the strongest fluorescent brightness.

### 3.3. Effect of Experimental Parameters on Fe^3+^ and Pb^2+^ Adsorption Capacity

#### 3.3.1. Effect of Contact Time

**Figure 5:**
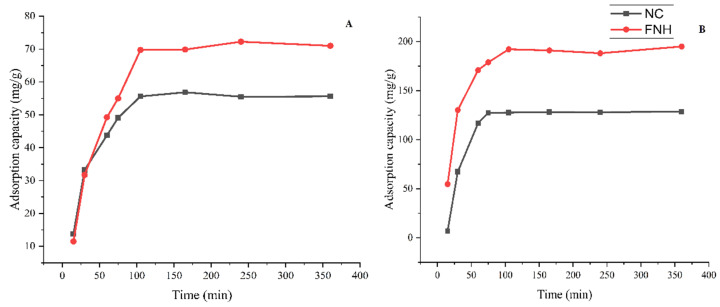
Effect of contact time on Fe^3+^ (**A**) and Pb^2+^ (**B**) adsorption.

Contact time was an important parameter in adsorption experiments. The effect of contact time on the adsorption capacity is shown in Figure 5. From 0–30 min, the adsorption capacity increase rapidly when contact time increased, which might be due to the more abundant adsorption sites on NC and FNH for Fe^3+^ and Pb^2+^ to occupy when time extended. Fe^3+^ and Pb^2+^ adsorption rate onto NC and FNH gradually decreased during 30–105 min and reached equilibrium gradually after 105 min. Therefore, the contact time was set at 105 min.

Meanwhile, the adsorption capacities of NC and FNH were compared (shown in Figure 5), which indicated FNH had a higher adsorption capacity (71.0 mg/g) toward Fe^3+^ than NC (55.6 mg/g), and a higher adsorption capacity (195.0 mg/g) toward Pb^2+^ than NC (128.5 mg/g). Thus, FNH was used as adsorbent in the following study.

#### 3.3.2. Effect of pH

The pH was a key parameter for affecting the adsorption capacity of FNH, since the state of metal ions differently under different H^+^ concentration, which might affect the interaction with the adsorbent.

**Figure 6:**
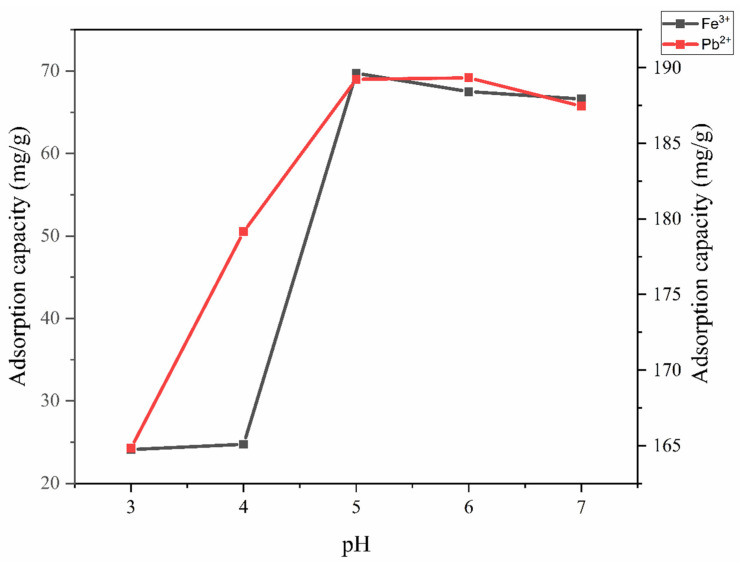
Effect of pH on Fe^3+^ and Pb^2+^ adsorption.

The precipitation will be formed when the pH of Fe^3+^ and Pb^2+^ solution was alkaline, so the pH of Fe^3+^ and Pb^2+^ solution was controlled in the range of 3.0–7.0. Figure 6 shows the influence of pH on metal removal using FNH materials. At high proton concentration (pH 3.0), the sorption capacity of the two materials for heavy metal ions was poor. This effect might be caused by H^+^ occupying the adsorption sites of FNH at low pH; in an acidic environment, the reactive groups on FNH (carboxylic/amino groups mainly) will be protonated, which weakens the sorbate/sorbent interactions and decreases the sorption efficiency [33,34]. The adsorption capacity of FNH to Fe^3+^ and Pb^2+^ increases with the increase of pH, and the adsorption capacity was the maximum at pH 5.0. Therefore, subsequent experiments were performed at pH 5.0.

#### 3.3.3. Effect of Adsorbent Dosage

**Figure 7:**
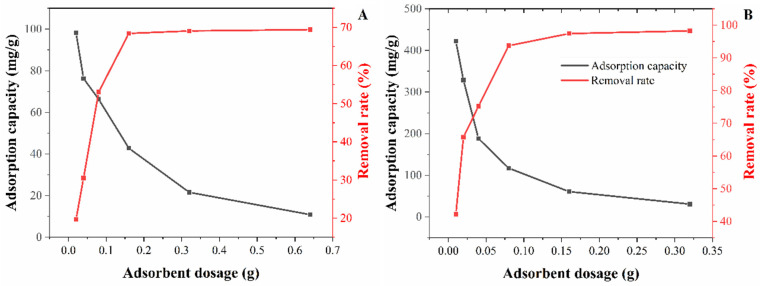
Effect of absorbent dosage on Fe^3+^ (**A**) and Pb^2+^ (**B**) adsorption.

The experimental results of the effect of adsorbent dosage on Fe^3+^ and Pb^2+^ adsorption in water was shown in Figure 7. When the dosage of the CNC-CDs in FNH increases from 0.02 g to 0.64 g, the removal rate of Fe^3+^ and Pb^2+^ significantly increase from 19.7% to 69.4% and from 42.2% to 98.2%, while the adsorption capacity dramatically drops from 98.3 mg/g to 10.8 mg/g and from 442.0 mg/g to 30.7 mg/g, respectively. As the amount of adsorbent dosage increases, the removal rate of Fe^3+^ and Pb^2+^ also increases. Since more adsorbent dosage provides more active adsorption sites, but at the same time, the adsorption capacity decreases, mainly due to the unsaturated adsorption of the adsorbent. To make better use of FNH, adsorbents loaded with 0.16 g and 0.08 g CNC-CDs in FNH were selected for subsequent adsorption experiments, respectively.

#### 3.3.4. Adsorption Isotherm

To study the isotherm during FNH adsorption of Fe^3+^ and Pb^2+^, experiments were performed at different initial Fe^3+^ and Pb^2+^ concentrations by adding FNH to Fe^3+^ and Pb^2+^ solutions (50 mL, pH 5). As the temperature increased from 303 K to 323 K, the adsorption capacity of FNH for Fe^3+^ and Pb^2+^ increased from 29.9 mg/g to 32.7 mg/g and from 29.9 mg/g to 32.7 mg/g, respectively. Meanwhile, the adsorption equilibrium data of FNH adsorption Fe^3+^ and Pb^2+^ were analyzed using the Langmuir and Freundlich isotherm models, which can be expressed by Equations (1) and (2):

*C_e_/q_e_* *=* 1/*K_L_q*_max_ *+* *C_e_/q*_max_ (1)

lg*q_e_* = lg*K_F_* + 1/nlg*C_e_* (2)

where *q_e_* is the adsorption capacity of the metal ion at equilibrium (mg/g), *C_e_* is the concentration of the metal ion in the aqueous solution at equilibrium (mg/L), *q_m_* and *K_L_* are the Langmuir constants related to the saturated adsorption capacity and binding energy, respectively, and *K_F_* and n are the Freundlich constants related to adsorption capacity and adsorption strength, respectively. In Table 1, *q_cal_*_,max_ and *q_exp,max_* are the adsorption capacities determined by theoretical calculations and experiments, respectively.

**Figure 8:**
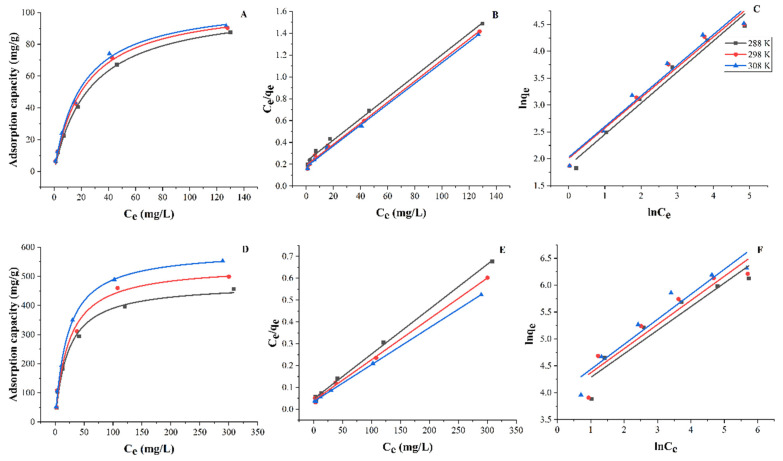
Adsorption isotherms of Fe^3+^ (**A**–**C**) and Pb^2+^ (**D**–**F**) and curves fitted by the Langmuir and Freundlich equation model.

The adsorption isotherm can be used to describe the interaction pattern between the adsorbate and the adsorbent, which can explain the adsorption isotherm mechanism. The Freundlich and Langmuir models were used to fit the isotherm adsorption properties of the FNH, and the fitting curve results are shown in Figure 8, and Table 1 and Table 2.

The Langmuir model is designed based on the monolayer adsorption process that the adsorption sites on the adsorbent surface are homogeneous and there is no interaction between adsorbates. From the data of the two models in Figure 8, and Table 1 and Table 2, the correlation coefficient of the Langmuir model is significantly higher than that of the Freundlich model; therefore, the Langmuire model is chosen for the interpretation of the adsorption capacity of the metal ion. The correlation constant (R^2^) of Langmuire model is greater than 0.980, indicating that the calculated value of the theoretical adsorption capacity will fit with the experimental value of adsorption. Thus, the adsorption of heavy metals by FNH might be monolayer adsorption.

### 3.4. Fluorescence Sensing of Fe^3+^

**Figure 9:**
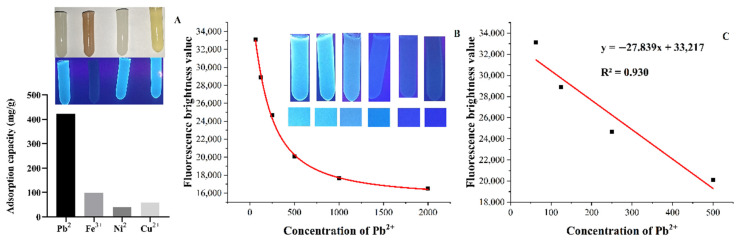
The adsorption capacity and fluorescence behavior of Pb^2+^, Fe^3+^, Ni^2+^, and Cu^2+^ (**A**). The relationship between the fluorescence behavior of FNH and the gradient fluorescence quenching of Fe^3+^ concentration (**B**). (**C**) shows the standard curve.

FNH was allowed to act as an optical sensor to achieve a fast visual response depending on the fluorescence quenching effect of Fe^3+^ on CDs. The sensitivity of FNH to detect Fe^3+^ concentration is shown in Figure 9B. With the double dilution of Fe^3+^ concentration (2000, 1000, 500, 250, 125, and 62.5 mg/L), the fluorescence color of FNH gradually changed from dark black to blue. The fluorescence color of FNH was quantified by the ImageJ software, with the concentration of the Fe^3+^ as abscissa and the brightness value as the ordinate, and the standard curve is calculated and shown in Figure 9C. The fitting equation and correlation coefficient of theFe^3+^ response curve was y = −27.839x + 33,217 and R^2^ = 0.930, respectively, with a calculated detection limit of 62.5 mg/L. The established equation confirms that the exponential function of the brightness value of FNH and Fe^3+^ concentration can be used for the determination of the Fe^3+^ concentration. The selective evaluation results of FNH on the adsorption capacity and fluorescence quenching performance of other heavy metal ions (Pb^2+^, Fe^3+^, Ni^2+^, and Cu^2+^) are shown in Figure 9A. The ability of FNH to adsorb and remove Pb^2+^ was the highest, followed by Fe^3+^. But in the visual reaction of fluorescence quenching effect, the fluorescence quenching reaction could only occur in the presence of Fe^3+^.

CNC itself was a natural adsorption-aggregator, which has outstanding performance in the removal of heavy metals. At the same time, the amino, hydroxyl, and carboxyl groups contained in FNH also provided heavy metal adsorption sites, which improved the adsorption and removal ability of heavy metals. The three-dimensional network structure of CNC-based fluorescent nanocellulose hydrogels provided sufficient ion transport channels to facilitate the diffusion of heavy metal ions from the outside to the inside of the adsorption site. The fluorescence excitation of CDs was limited, resulting in a fluorescence quenching effect, which reduced the fluorescence brightness of FNH.

## 4. Conclusions

In summary, to better purify heavy metal ions in water, a CDs-based FNH with high-performance luminescence properties was prepared. In the preparation of FNH, nanocellulose (NC) needed to be carboxylated first to obtain carboxylated nanocellulose (CNC), then the CDs were grafted onto nanocellulose by the EDC/NHS coupling method, and, finally, the fluorescent nanocellulose hydrogel was obtained using the traditional free radical polymerization method. A novel FNH based on carboxylated nanocellulose (CNC) grafted carbon dots (CDs) was successfully synthesized by the above method, which could be used as biosorbent and biosensor for heavy metal removal and detection. After NC was carboxylated and grafted, the number of carboxyl and amino groups that can adsorb heavy metal ions increased, so the adsorption capacity of FNH to heavy metal ions was also greatly improved compared with NC. The removal rate of Fe^3+^ and Pb^2+^ by FNH exceeded 69.4% and 98.2%, and the adsorption capacity amount reached 98.3 mg/g and 442.0 mg/g.

## Figures and Tables

**Table 1:** Isothermal parameters obtained by the Langmuir model fitting.

Temperature		R^2^	*q_cal,mas_* (mg/g)	*q_exp,max_* (mg/g)	*K_L_* (L/mg)
288 K ^a^	Fe^3+^	0.9981	86.66	87.50	0.0377
298 K		0.9975	90.24	90.25	0.0446
308 K		0.9967	91.76	91.50	0.0491
288 K	Pb^2+^	0.9872	443.8	456.3	0.0460
298 K		0.9830	492.0	498.8	0.0439
308 K		0.9975	549.3	552.5	0.0473

^a^ K: Thermodynamic temperature unit (Kelvins), with absolute zero as the starting point for calculation, every 1K change is equivalent to a 1 °C change (0 °C ≈ 273 K).

**Table 2:** Isothermal parameters obtained by the Freundlich model fitting.

Temperature		R^2^	n	*K_L_*
288 K	Fe^3+^	0.9594	2.232	10.42
298 K		0.9443	2.341	12.09
308 K		0.9324	2.393	12.94
288 K	Pb^2+^	0.9287	3.068	75.57
298 K		0.9071	3.046	84.06
308 K		0.9051	3.031	92.67

## Data Availability

Data is contained within the article.
